# Channel reconstruction and dual attention dynamic fusion for remote sensing image semantic segmentation

**DOI:** 10.1371/journal.pone.0343777

**Published:** 2026-03-20

**Authors:** Xin Wang, Longxing Niu, Zhiwen Zheng, Qun Yang, Jia Lu, Hao Yang, Qin Qin, Guan Lian, Jiawei Wang

**Affiliations:** 1 School of Computer and Information Security, Guilin University of Electronic Technology, Guilin, China; 2 School of Computer Engineering, Guilin University of Electronic Technology, Beihai, China; 3 School of Information and Software Engineering, University of Electronic Science and Technology of China, Chengdu, China; 4 Guilin Blue Harbor Technology Co., Ltd, Guilin, China; 5 School of Electronic Information, Guilin University of Electronic Technology, Beihai, China; 6 School of Architecture and Transportation Engineering, Guilin University of Electronic Technology, Guilin, China; 7 Dakang Supply Chain (Guangxi) Group Co., Ltd., Nanning, China; Shandong Agricultural University, CHINA

## Abstract

As the spatial resolution of remote sensing imagery continues to be improved, the complexity of the information also increases. Remote sensing images generally have characteristics such as wide imaging ranges, dispersed distribution of similar land objects, complex boundary shapes, and dense small targets, which pose severe challenges to semantic segmentation tasks. To address these challenges, we propose a channel reconstruction and dual attention dynamic fusion network (CRDFNet), which is a semantic segmentation network for remote sensing image that can effectively integrate global and local contexts. To better handle complex boundary shapes, we designed a channel feature aggregation module (CFAM), which can extract spatially redundant information during feature fusion and enhance high-resolution detail features. Through a channel reconstruction block, it promotes the alignment of fine-grained information from the encoder with high-level semantic information from the decoder, effectively aggregating multi-scale features extracted by the encoder and significantly improving segmentation accuracy. At the same time, to optimize the segmentation performance of small targets, we propose a dual attention feature refinement module (DAFRM), which achieves precise segmentation of small targets by effectively fuses the shallow spatial features of the encoder and the deep semantic features of the decoder through a dynamic fusion mechanism guided by dual attention. Experimental results on the Potsdam, Vaihingen, UAVid, and MSIDBG datasets demonstrate that CRDFNet outperforms existing methods in terms of F1 score, OA, and mIoU (Intersection over Union), validating its excellent performance.

## 1. Introduction

With the advancement of remote sensing technology, we are now able to obtain a large number of high-resolution, multispectral remote sensing images. These images contain rich surface information, and high-quality semantic segmentation has become a highly focused task in remote sensing image processing, widely applied in fields such as environmental monitoring, urban planning, and land resource utilization [1–3]. Remote sensing images are characterized by complex texture information, disordered category distributions, and challenges like scale diversity [4,5]. Despite the progress in semantic segmentation technology, which provides new opportunities for understanding and automating the annotation of ultra-high-resolution images, developing more efficient and accurate segmentation algorithms remains a key issue in current remote sensing image processing.

As convolutional neural networks (CNNs) have been successfully and organically integrated into an increasing number of tasks, they have demonstrated exceptional feature extraction and model representation capabilities in remote sensing semantic segmentation tasks [6–8]. Influenced by research in remote sensing image semantic segmentation, the encoder-decoder architecture has gradually become the recognized framework for this task [9,10]. This architecture continuously downsamples images during the encoding phase to gather detailed information, and then performs continuous upsampling during the prediction phase to reconstruct the image. Many works, such as UNet [11] and DeepLabv3 [12], have achieved impressive results based on this architecture, making encoder-decoder networks the mainstream paradigm in remote sensing image semantic segmentation [13,14].

However, due to the limited receptive field of CNNs, their ability to process contextual information is somewhat restricted. This often leads to inaccurate feature extraction for specific targets (such as buildings, especially small-scale ones), and related information can easily be lost as the network deepens [15,16]. In response to this, Li et al. [17] designed a multi-scale spatial attention module aimed at dynamically supplementing contextual information extracted by the model, enhancing its ability to perceive targets of different scales effectively. With the introduction of the transformer model [18–21], while it offers a way to capture global information, its performance in extracting local information is relatively weak, resulting in less detailed segmentation outcomes. As a result, the current research trend tends to combine CNNs with transformer models to leverage their strengths in capturing both local and global image features [22–25]. For instance, the UNetFormer model proposed by Wang et al. [26] merges the UNet structure with a CNN-based encoder and a transformer-based decoder, showing significant advantages in remote sensing land cover extraction in urban environments. Meanwhile, TransUNet by Chen et al. [27] extracts local features through extensive convolutions and integrates multiple transformer modules to construct a global context model. By designing cross-stage fusion modules, it effectively combines both local and global feature information.

Global and local information play a crucial role in accurately understanding the semantic structure of an image [28–30]. Although transformer-based methods have shown good results, they are relatively slow in expanding the receptive field and require stacking a large number of modules to achieve global self-attention, thereby increasing computational complexity and overhead [31,32]. Additionally, these methods have certain limitations in modeling local visual structures and scale representation, especially in maintaining the integrity of object boundaries and accurately identifying small targets.

Based on these challenges, this paper proposes channel reconstruction and dual attention dynamic fusion network (CRDFNet), which significantly reduces computational costs while maintaining high segmentation accuracy for high-resolution image semantic segmentation. The main contributions are summarized as follows:

- We propose CRDFNet with an encoder-decoder structure. This network extracts and aggregates multi-scale information in the CNN-based encoder, and then performs feature decoding by the Transformer based decoder. A feature refinement mechanism is introduced during the decoding process to effectively integrate global and local context information. Thereby enhancing the representational ability and segmentation accuracy of semantic information.
- We propose a channel feature aggregation module (CFAM), which employs an adaptive channel redistribution mechanism and multi-scale feature interaction to effectively aggregate multi-scale features extracted by the encoder, enhancing the model’s feature representation capabilities at different scales to improve segmentation performance and detail capture in complex scenes.
- We propose a dual attention feature refinement module (DAFRM), which effectively integrates the fine-grained spatial information of the encoder with the advanced semantic context of the decoder through a dynamic fusion mechanism guided by dual attention. This enhances the feature representation of small targets and significantly improves their segmentation accuracy.

The remainder of this paper is organized as follows. Section 2 introduces related work on remote sensing image segmentation. Section 3 explains the proposed method. Sections 4 and 5 present the experimental results and conclusions, respectively.

## 2. Related work

### 2.1. CNN-Transformer hybrid architecture

With the rapid development of deep learning [33,34], convolutional neural networks (CNNs) have become a core technology in the field of computer vision, especially in semantic segmentation tasks, where the application of CNNs has significantly improved both performance and efficiency. The introduction of fully convolutional networks (FCN) is widely regarded as pioneering work in applying deep learning to semantic segmentation [35]. The key innovation of FCN lies in replacing traditional fully connected layers with convolutional layers, allowing the network to handle input images of arbitrary size while effectively preserving spatial structural information. Subsequently, various CNN-based innovative networks have been proposed, such as UNet [11], SegNet [36], PSPNet [37], and DANet [15]. However, these networks lose contextual information during the downsampling and upsampling processes, leading to a decrease in segmentation accuracy. In recent years, the transformer architecture has been successfully extended to computer vision, especially in semantic segmentation tasks, demonstrating exceptional modeling capabilities. For example, SETR [38] was the first to adopt a pure transformer structure, replacing the traditional CNN encoder to achieve comprehensive modeling of global features. However, when applied to image tasks, the computational complexity of transformers grows quadratically with the input resolution, which greatly limits their application in ultra-high-resolution image segmentation. To address this challenge, swin transformer [39] introduced sliding windows and hierarchical structures, effectively reducing computational complexity while maintaining global context modeling capabilities. As research has deepened, the combination of CNN and transformer has gradually become mainstream, and improved versions of swin transformer have been widely applied in fields such as remote sensing image segmentation. Chen et al. [40] pointed out that although the enhanced ViT architecture excels in modeling long-range dependencies, it often overlooks local spatial features. To solve this issue, Zhang et al. [41] proposed a hybrid framework, using swin transformer as the encoder to capture global dependencies, while the decoder utilizes CNNs to preserve local information. Wu et al. [42] ingeniously combined the strengths of CNN and transformer by extracting local features using ResNet50 and capturing global information with transformer, particularly through a multi-scale multi-head self-attention mechanism that efficiently extracts multi-scale context information, enhances channel interactions with an efficient feed-forward neural network, and adapts the fusion of deep and shallow features through multi-scale attention. This approach achieved outstanding segmentation performance on the ISPRS dataset while maintaining low computational complexity, especially excelling in handling small objects and complex scenes. Thanks to these advancements, we propose a remote sensing image semantic segmentation method that combines CNNs and transformers.

### 2.2. Multi-scale feature fusion for Semantic Segmentation

In high-resolution remote sensing images, there are significant variations in object scales, and effectively integrating the multi-scale features extracted by the encoder is key to improving segmentation accuracy. To address this, researchers often introduce attention mechanisms into base models to enhance feature fusion. For instance, Li et al. [43] designed a dedicated attention aggregation module that effectively solves the multi-scale feature fusion problem in fine-grained remote sensing image semantic segmentation, while maintaining low computational complexity. BASNet, proposed by Qin et al. [44], processes the multi-scale features extracted by the encoder through a residual refinement module, enhancing feature representation capabilities and significantly improving boundary segmentation performance. Li et al. [45] further innovated by proposing a parallel multi-level feature enhancement group and a feature-weighted fusion module, which integrates multi-scale features from the encoder, addressing challenges such as large variations in object scales, loss of details, and semantic gaps in remote sensing images. This significantly enhances the model’s ability to represent multi-scale features and demonstrates strong robustness when handling challenging scenarios like complex architectural structures and shadow occlusions. Inspired by the above studies, we have designed a channel feature aggregation module to efficiently integrate multi-scale features extracted by the encoder, further improving the model’s feature representation capability at different scales, thereby enhancing its segmentation performance and detail capture ability in complex scenes.

### 2.3. Attention-based feature refinement for Semantic Segmentation

To achieve the adaptive extraction and enhancement of task-related features, various attention mechanisms have been widely applied in the field of semantic segmentation. DANet [15] designs a dual-attention network that incorporates two parallel attention modules: channel attention and position attention, aiming to simultaneously model the global feature dependencies in these two dimensions. Specifically, the position attention module focuses on learning the spatial interdependencies of the feature map, while the channel attention module models the intrinsic relationships between different channels. By building rich contextual dependencies on top of local features, DANet significantly improves segmentation accuracy. He et al. [46] use high-level semantic features from deep layers of the encoder to generate class activation maps, modeling intra-class (different scales of the same category) and inter-class (different categories) scale variations, forming a dual-path attention map. This attention map is fused with the feature map containing fine-grained details, selectively enhancing discriminative features and suppressing redundant information, effectively alleviating the boundary blurring caused by multi-scale fusion. Zhang et al. [47] introduce a blur-aware attention mechanism and employ a two-stage feature weighting strategy, achieving progressive feature optimization from blurred area localization to local detail enhancement, significantly improving feature clarity. Based on these studies, we have designed a dual-attention feature refinement module that efficiently fuses shallow spatial information with deep semantic information through a dynamic fusion mechanism, significantly enhancing the model’s accuracy in processing details and small objects.

## 3. Materials and methods

### 3.1. Overview structure

**Fig 1:**
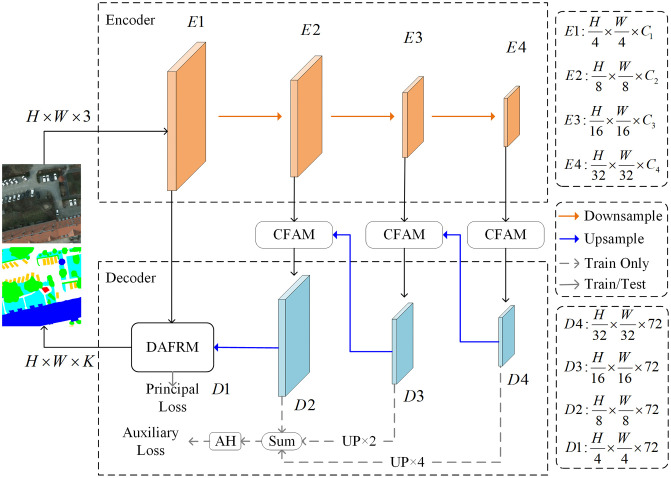
The overall architecture of CRDFNet.

The channel reconstruction and dual attention dynamic fusion network (CRDFNet) proposed in this paper adopts a classic encoder-decoder architecture, and the overall network structure is shown in Fig 1. The encoder uses RegNet [48] as the backbone network, responsible for extracting multi-scale feature maps at four levels: E1, E2, E3, and E4. The decoder is built around three global-local attention blocks (GLTB) [26], which progressively decode the deep features output by the encoder, generating the corresponding features D4, D3, and D2. To facilitate full information interaction between the encoder and decoder, the feature layers E2, E3, and E4 establish skip connections with the corresponding stages of the decoder through an efficient channel feature aggregation module (CFAM), achieving effective fusion of features at different levels. This module filters out spatial redundant information during the feature fusion process, enhances high-resolution detailed features, and then uses channel reconstruction blocks to achieve precise alignment of fine-grained information from the encoder and advanced semantic information from the decoder. Finally, the shallow encoder feature E1 and the deep decoder feature D2 are input together into the dual attention feature refinement module (DAFRM) for final adaptive decoding. This module leverages channel and spatial attention mechanisms to enhance the shallow details and recalibrate the deep channel weights, respectively. It then achieves adaptive feature fusion through global channel-guided dimensionality reduction and spatial dependency modeling, ultimately producing the final semantic segmentation result. To ensure model training stability and performance, CRDFNet employs a supervision strategy that combines primary and auxiliary losses. The primary loss is applied to the final output of DAFRM. At the same time, to enhance feature learning at intermediate layers of the network, auxiliary loss is introduced. Specifically, the D4 feature map is upsampled by a factor of 4, and the D3 feature map is upsampled by a factor of 2, then they are directly added to the D2 feature map to jointly compute the auxiliary loss. This meticulously designed architecture enables CRDFNet to achieve high-precision feature representation and efficient encoding-decoding processes while ensuring low computational overhead, demonstrating excellent segmentation performance.

### 3.2. Efficient CNN-based encoder

The CNN-based encoder is capable of extracting multi-scale local features, which are progressively extracted in a hierarchical manner from low-level to high-level modules, enabling effective analysis of deep features. Ilija Radosavovic et al. [48] proposed the RegNet architecture, which innovatively introduced the concept of a “design space” By exploring a structured network design space, they discovered a series of network architectures that not only provide similar or even better performance but also significantly reduce computational costs, thereby achieving higher efficiency and faster inference speed. The RegNet architecture is similar to ResNet [49] and guides the design of network width and depth through quantized linear functions. Its structure initially extracts features through a 3 × 3 convolution layer combined with batch normalization (BN) and a ReLU activation function. This is followed by four stages, each of which reduces the height and width of the input feature map by half. Each stage consists of multiple stacked blocks, where the first block includes a group convolution with stride 2 (main branch) and a standard convolution (shortcut branch), while the convolutions in the remaining blocks use a stride of 1. Finally, features are output through a global average pooling layer and a fully connected layer. In the CRDFNet designed in this paper, we constructed an encoder containing four levels of RegNet modules, corresponding to the network’s E1, E2, E3, and E4 output layers. These modules are capable of providing rich feature maps originating from different receptive fields.

### 3.3. Transformer-based decoder

**Fig 2:**
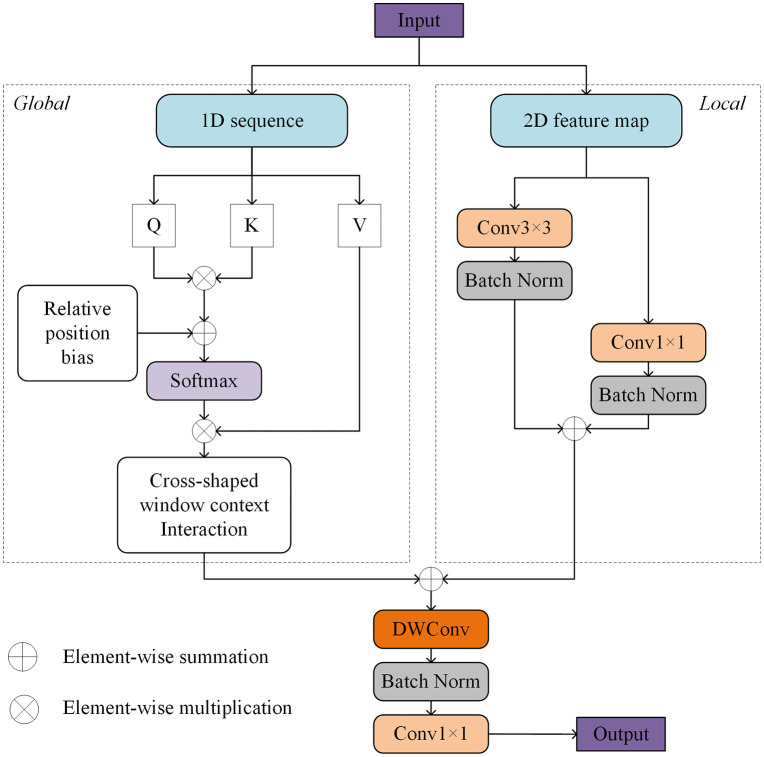
Decoder based on Transformer.

To address the refined segmentation needs of urban remote sensing images with complex backgrounds, this paper proposes a transformer decoder with a dual-branch parallel structure, like the introduction in UnetFormer [26], and the structure is shown in the Fig 2. Through a collaborative mechanism of global perception and local enhancement, we achieve multi-scale feature modeling. The architecture employs a global branch that utilizes a window-based multi-head self-attention mechanism to capture long-range global dependencies. By partitioning the feature map into multiple non-overlapping windows and performing attention computation within each window, computational complexity is reduced. Additionally, a cross-shaped window context interaction module is introduced to enhance information exchange between windows and improve global modeling capabilities. Specifically, the cross-shaped window mechanism performs pooling of window features along the horizontal and vertical directions, establishing dependencies among windows in the corresponding directions. The pooled results from both directions are then fused to form a “cross-shaped” context interaction map, capturing long-range dependencies between windows. Additionally, we introduce relative position bias to optimize spatial relationships and further improve the model’s ability to understand and capture spatial structures. The local branch is designed to extract local features using dual-path convolutions of 3 × 3 and 1 × 1, combined with batch normalization to enhance training stability. The features from both branches are efficiently fused through depthwise separable convolutions and batch normalization, thus retaining the advantages of the transformer in global modeling while enhancing local sensitivity through convolutional priors. Ultimately, while ensuring high-resolution spatial details, this method effectively improves the accuracy of object boundary recognition.

### 3.4. Channel feature aggregation module

**Fig 3:**
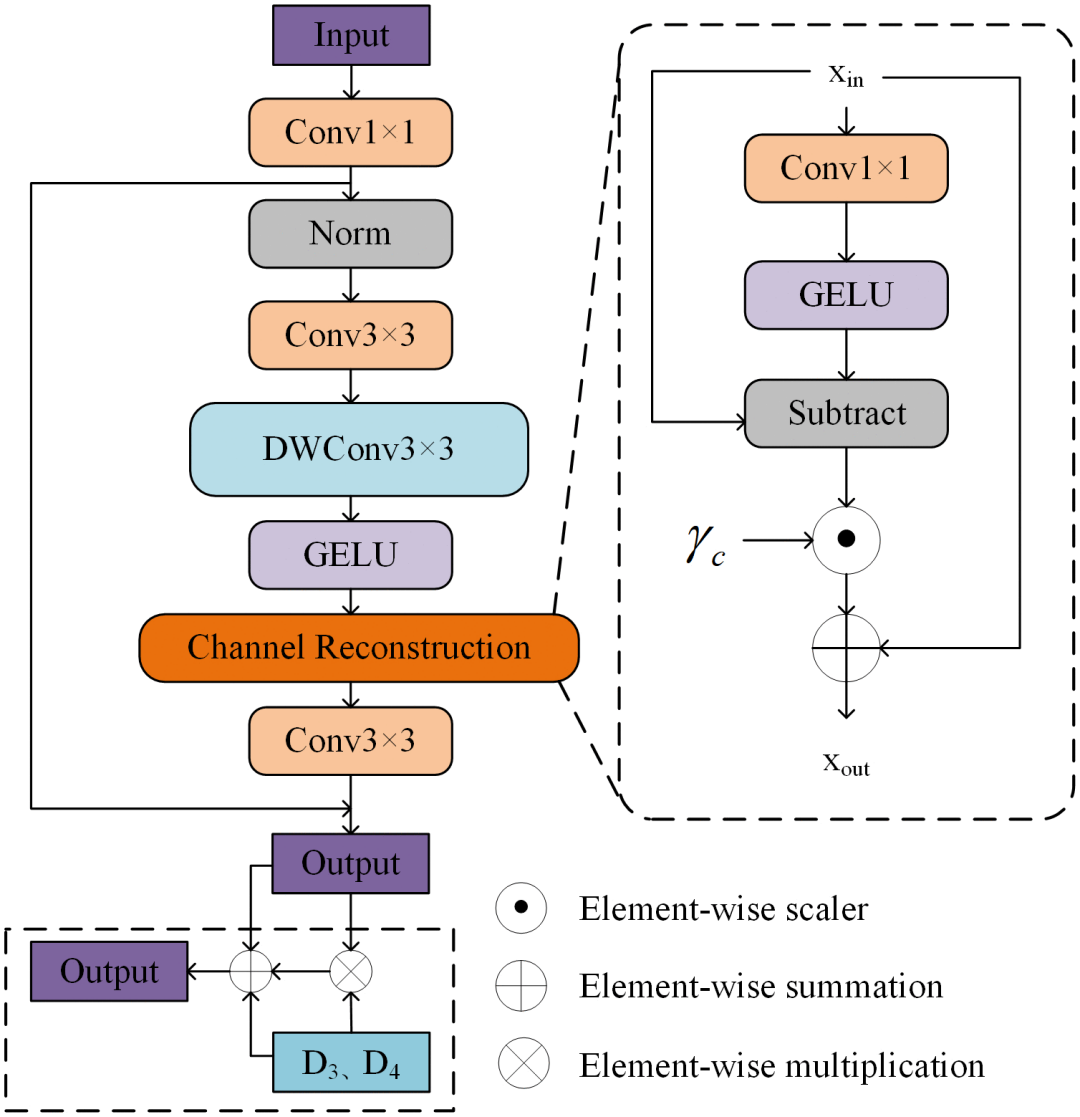
The detailed architecture of the CFAM.


In remote sensing image semantic segmentation, the effective fusion of multi-scale features extracted by the encoder—where deep features are rich in semantic meaning and shallow features abound in spatial details—is crucial. However, simple feature stacking fails to distinguish feature importance, while the spatial redundancy in shallow features can adversely affect the fusion outcome. To effectively fuse these multi-scale features and optimize their representation, this paper designs a channel feature aggregation module (CFAM). The structure of the CFAM is illustrated in Fig 3. The core function of this module is to adaptively suppress spatial redundant information in the local features that is irrelevant to the semantic segmentation task, while significantly enhancing high-resolution channel features that are crucial for recovering image details and precise boundaries. CFAM has the ability of “adaptive channel reallocation” and “enhanced channel interaction,” meaning it can optimize and reorganize the local information extracted by the encoder. Through this approach, the model can learn not only which features are most important during the fusion process, but also how different scale features should collaborate, rather than simply stacking features together. The process is as follows: the features extracted by the encoder are input into CFAM, first passing through a 1 × 1 convolutional layer for feature extraction and adjusting the number of input channels. Then, the output of the convolution is normalized to accelerate model training and improve stability. Next, the features are passed through a 3 × 3 convolutional layer to further extract richer features, followed by a 3 × 3 depthwise separable convolutional layer that applies convolution to each channel individually, reducing computational cost. Afterward, a GELU activation function is applied to enhance the network’s nonlinear expression capability. Finally, a channel reconstruction (CR) block is used to optimize the interactions at the channel level, significantly improving the alignment and coordination of the fine spatial information from the encoder with the high-level semantic information in the decoder. This block reallocates channel features through channel reduction projection (*Wr*) and GELU activation, as defined by the following formula:

$$CR\left(X\right)=X+\gamma_{c}⊙\left(X-GELU\left(XW_{r}\right)\right),$$ (1)

where *X* is the input feature, and $\gamma_{c}$ is a channel scaling factor initialized to zero. It is capable of capturing and expressing complex and useful interaction relationships between channels, thereby enhancing the overall feature representation capability of the model. Finally, another 3 × 3 convolution is applied to further integrate the features. The feature *F*_*C*_ is then combined with the upsampled *D*_*4*_ and *D*_*3*_ features from the decoder D4 and D3 through channel multiplication, resulting in weighted features, followed by a residual connection to output the final feature *F*_*C-OUT*._ The formula is as follows:


$$F_{C-OUT}=F_{C}\times D_{i}+F_{C}+D_{i},$$ (2)

where *i* is the 3 or 4.

Although the SE module and its variants (such as CBAM) have been widely used for channel-wise feature recalibration, the Channel Reconstruction (CR) block of CFAM differs fundamentally from them in both structure and objective. The SE module learns channel weights through global pooling and fully connected layers, focusing on inter-channel dependencies. In contrast, the CR block aligns fine-grained encoder information with high-level semantic decoder information via learnable channel scaling factors $\gamma_{c}$ and a residual reconstruction mechanism. Its goal is not only to recalibrate channels but also to facilitate structural alignment and complementary information integration across layers. Furthermore, CFAM employs multi-scale convolutions and depthwise separable convolutions for local feature extraction and interaction, enhancing the preservation of spatial details—a capability not present in the SE module.

CFAM through adaptive learning, fully explores fine-grained information such as edges and textures contained in the encoder, and efficiently fuses this with high-level semantic features such as “buildings” or “roads” in the decoder. With its unique channel reallocation mechanism, CFAM enhances the discriminative power of the fused features. For example, when performing fine classification of objects like building roof materials or different types of vegetation, this module strengthens the channel information that helps distinguish subtle differences, thereby improving segmentation accuracy.

### 3.5. Dual attention feature refinement Module

**Fig 4:**
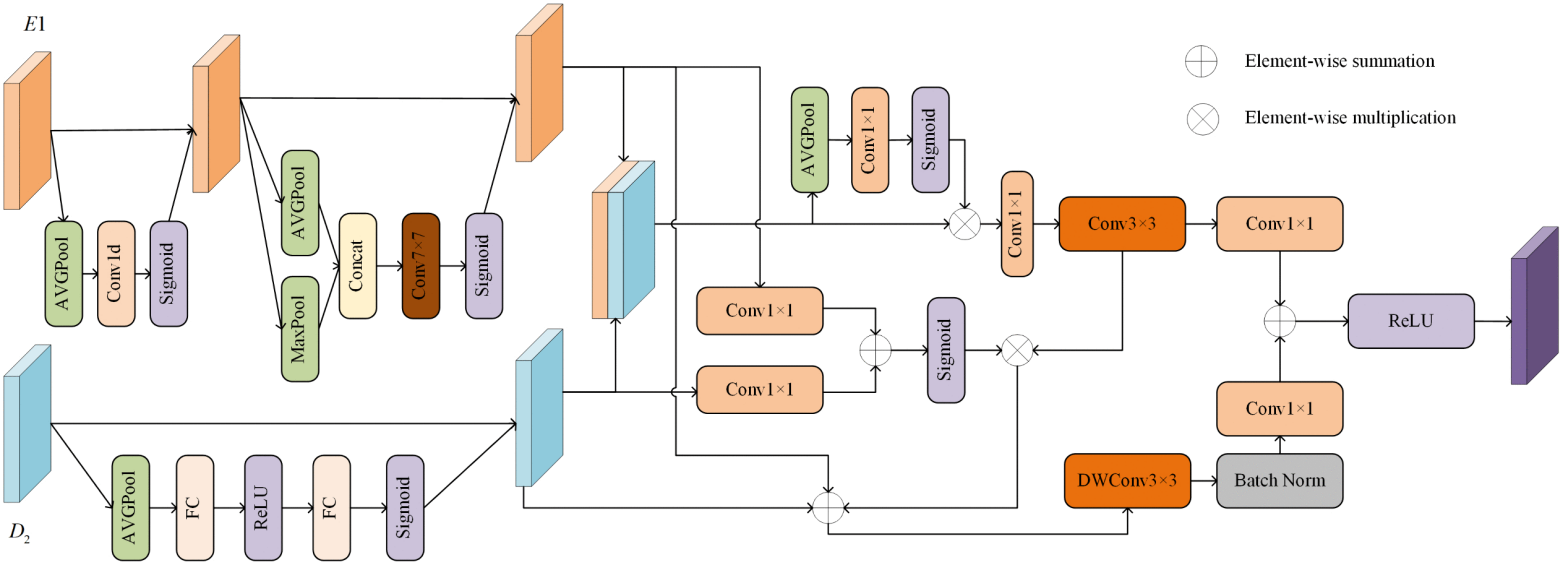
The detailed architecture of the DAFRM.


In remote sensing image semantic segmentation, the problem of detail loss and poor small target segmentation performance due to feature fusion mismatch exists. To address this issue, we designed the dual attention feature refinement module (DAFRM). This module employs a dual-attention mechanism to achieve the complementarity and enhancement of semantic information and spatial details. The channel attention mechanism identifies and strengthens key detail channels in shallow-layer features, while the spatial attention mechanism focuses on important positional regions. Building on this, a globally guided dynamic fusion strategy effectively improves the model’s feature utilization efficiency and segmentation accuracy. As shown in Fig 4, we perform channel attention processing on *E1* to identify and enhance key feature channels. Specifically, by combining global average pooling and 1D convolution operations, the network can learn the importance of each feature channel and assign corresponding weights. The weighting of key channels significantly enhances the discriminative power of the feature maps generated by the network. Notably, the introduced 1D convolution not only effectively captures correlations between local channels but also significantly reduces the number of parameters and computational overhead. Compared to fully connected layers, it avoids potential overfitting risks. The formula is as follows:

$$w_{e-ch}=Sigmoid\left(Conv1d\left(AVGPool\left(E1\right)\right)\right),$$ (3)

where *w*_*e-ch*_ represents the channel weights of the original feature map *E1*, which are multiplied with *E1* to obtain the enhanced feature map *E1’*. *Conv1d* represents 1D convolution. To further enhance spatial information, we introduce a spatial attention mechanism. The feature map, after channel attention processing, undergoes global average pooling and maximum pooling operations, with the results concatenated along the channel dimension. Next, a 7 × 7 2D convolution processes the concatenated two-channel feature map, mapping it to a single-channel raw spatial attention map. Finally, after normalization through the Sigmoid activation function σ, the final spatial attention map *w*_*e-sp*_ is obtained, which is then multiplied with the feature map *E1’* through channel multiplication to produce the feature map *F*_*E*_. The formula is as follows:


$$\begin{array}{c} w_{e-sp}=Sigmoid\left(Conv_{\text{7}}\left(\left[AVGPool\left(E1\text{'}\right);MaxPool\left(E1\text{'}\right)\right]\right)\right), \end{array}$$ (4)

where *Conv*_*7*_ represents a 2D convolution with a kernel size of 7. Through the aforementioned stacked attention mechanism, we have achieved enhanced depth details in the shallow feature map *E1*.

The feature map *D*_*2*_ obtained from upsampling the decoder D2 is processed with channel squeezing and excitation. This attention mechanism is specifically designed for the channel dimension. The core idea is to enable the network to adaptively learn the importance of each channel, thereby assigning weights to each channel in the feature map, emphasizing useful features, suppressing irrelevant features, enhancing the feature expression capability, and boosting semantic information. The process is mainly divided into two steps: Squeeze and Excitation. The Squeeze operation integrates global information by performing global average pooling on each channel, compressing the *H×W* feature map to a 1 × 1 map to obtain the global statistic *z*_*c*_ for each channel. The formula is as follows:


$$z_{c}=\frac{\text{1}}{H\times W}\sum_{i=1}^{H}\sum_{j=1}^{W}u_{c\left(i,j\right)},$$ (5)

where *u*_*c(i,j)*_ is the value of the c-th channel at position *(i,j).*

The Excitation operation performs adaptive recalibration by weighting the channel descriptors *z* obtained from the Squeeze operation. This is done through a bottleneck structure containing two fully connected (FC) layers (first reducing the dimensionality and then increasing it), with a ReLU activation in between, and finally using Sigmoid normalization to obtain the weight *s*_*c*_ for each channel. The formula is as follows:


$$s=\sigma \left(W_{\text{2}}\cdot \delta \left(W_{\text{1}}z\right)\right)$$ (6)

where *δ* is ReLU, *σ* is Sigmoid, and *W*_*1*_ and *W*_*2*_ are the parameters of the fully connected layers. The final output is obtained by performing channel multiplication between *s*_*c*_ and the original feature map *u*_*c*_, resulting in the weighted feature map *F*_*D*_. The formula is as follows:


$$F_{D}=s_{c}\cdot u_{c}$$ (7)

The feature maps *F*_*E*_ and *F*_*D*_ mentioned above are concatenated along the channel dimension to form a feature map *F* with doubled channels. This concatenation integrates feature information from different paths, laying the foundation for subsequent dynamic fusion. To ensure that the fused features can be utilized by subsequent modules (often requiring the channel number to be restored to the original size), DAFRM adopts a channel dimension reduction mechanism guided by global channel information, rather than a simple 1x1 convolution. Firstly, the concatenated feature map *F* undergoes global average pooling to compress its spatial information into channel descriptors. Then, through a 1x1 convolutional layer and a Sigmoid activation function, channel weights *w*_*ch*_ are generated. The formula is as follows:


$$w_{ch}=Sigmoid\left(Conv_{\text{1}}\left(AVGPool\left(F\right)\right)\right)$$ (8)

where *Conv*_*1*_ represents a 2D convolution with a kernel size of 1. The obtained *w*_*ch*_ is used to calibrate the concatenated feature map *F*, and then another 1x1 convolutional layer is applied to select important feature maps and reduce the channel number to the original size C, generating the feature map *F*_*ch*_. This convolutional layer, guided by *w*_*ch*_, can selectively retain important features while discarding features with less information. A 3 × 3 convolution is then applied to *F*_*ch*_ to obtain the feature map *F*_*p*_, which effectively captures local features in the image. After fusion and dimensionality reduction along the channel dimension, the DAFRM further models the spatial dependencies between local feature maps. Spatial information is extracted from the original *F*_*E*_ and *F*_*D*_ through 1x1 convolutional layers, and the two are added together (element-wise summation). Finally, a sigmoid activation function is applied to generate spatial weights *w*_*sp*_, and the formula is as follows:


$$w_{sp}=Sigmoid(Conv_{\text{1}}\left(F_{E}\right)⊕Conv_{\text{1}}\left(F_{D}\right))$$ (9)

where ⊕ represents element-wise summation. *w*_*sp*_ describes the importance of each spatial position. The obtained spatial information *w*_*sp*_ is then used to calibrate the previously obtained feature *F*_*p*_, generating an adaptive fusion feature *F*_*af*_. This is combined with *F*_*E*_ and *F*_*D*_ through a residual connection. The resulting feature undergoes a 3 × 3 depthwise separable convolution followed by normalization to obtain the feature *F*_*d*_. This operation not only significantly reduces the number of parameters and computational cost but also maintains the model’s performance. Finally, *F*_*p*_ and *F*_*d*_ are separately processed through 1x1 convolutional layers to extract and handle channel dimension information, and the two are added together (element-wise summation). The result is passed through a ReLU activation function to obtain the final output feature map *F*_*out*_, with the formula as follows:


$$F_{out}=ReLU(Conv_{\text{1}}\left(F_{p}\right)⊕Conv_{\text{1}}\left(F_{d}\right))$$ (10)

Common adaptive feature fusion modules typically directly concatenate or sum the features to be fused, followed by adaptive recalibration. However, this “mix first, adjust later” strategy can lead to insufficient adaptability or feature confusion during fusion when the features differ significantly, such as shallow-level details and deep-level semantics. In contrast, DAFRM adopts a two-stage fusion paradigm of “enhancement and calibration first, dynamic selection later.” In the first stage, instead of direct fusion, we perform targeted preprocessing on features from different sources through a dual-path attention mechanism: channel and spatial attention are used to excavate and enhance the detailed information in shallow features E1, while channel compression and excitation mechanisms are employed to purify and reinforce the semantic discriminability of deep features D2. In the second stage, after feature concatenation, we introduce dynamic dimensionality reduction based on global channel guidance and dual-source spatial dependency modeling. This method not only performs simple weight multiplication but also allows the network to dynamically select and combine the most useful feature subsets from both paths under the guidance of global information, while integrating both detail and semantic information to refine spatial weights. Therefore, DAFRM achieves a deep fusion mechanism based on guidance and selection, with its core objective being to promote complementary synergy between the two types of features, rather than simple weighted blending. This design enables the model to demonstrate significant advantages over generic adaptive fusion modules in enhancing the visibility of small targets and the accuracy of boundary segmentation.

Through the above processing, DAFRM adaptively fuses the shallow spatial features from the encoder with the deep semantic features from the decoder. This not only effectively improves the model’s generalization ability when handling different remote sensing datasets but also significantly enhances its robustness in complex scenarios. At the same time, it cleverly integrates multi-scale information, enabling the model to recognize both the overall contours of large objects and the fine structures of small targets, thereby significantly improving its ability to identify various types of objects and ensuring accurate segmentation of objects of different sizes. Furthermore, the dual attention mechanism introduced by DAFRM is particularly important. This mechanism allocates higher attention weights to key regions in the image (especially object boundaries and small structures), effectively guiding the model to perform more detailed processing. This greatly improves the boundary precision and detail recovery capability of the segmentation results, making the generated segmentation map edges sharper and more aligned with the actual object shapes.

### 3.6. Loss function

The loss function in this paper combines pixel-wise cross-entropy loss and region-wise dice loss as the primary losses to comprehensively measure segmentation accuracy. Additionally, a weighted auxiliary cross-entropy loss is introduced, applied to the intermediate outputs of the network to provide extra supervision signals, particularly for optimizing the learning of GLTB. This multi-loss function strategy aims to improve segmentation precision and robustness. The formula for the cross-entropy loss ${ℒ}_{ce}$ is as follows:


$${ℒ}_{ce}=-\frac{\text{1}}{N}\sum_{n=1}^{N}\sum_{k=1}^{K}y_{k}^{\left(n\right)}log{\hat{y}}_{k}^{\left(n\right)}$$ (11)

where *N* is the number of samples, *K* is the number of categories, $y_{k}^{\left(n\right)}$ is the k-th element in the one-hot encoding of the true semantic label for sample n, and ${\hat{y}}_{k}^{\left(n\right)}$ is the confidence score from the softmax output of the network predicting sample n belongs to category k.

Dice loss is a region similarity-based loss function, particularly suitable for handling class imbalance issues, as it directly measures the overlap between predicted segmentation results and ground truth labels. The dice loss ${ℒ}_{dice}$ formula is as follows:


$${ℒ}_{dice}=1-\frac{\text{2}}{N}\sum_{n=1}^{N}\sum_{k=1}^{K}\frac{{\hat{y}}_{k}^{\left(n\right)}y_{k}^{\left(n\right)}}{{\hat{y}}_{k}^{\left(n\right)}+y_{k}^{\left(n\right)}}$$ (12)

The main loss ${ℒ}_{main}$, by combining pixel-level cross-entropy loss and region-level dice loss, provides more comprehensive guidance for network learning, focusing not only on the correct classification of each pixel but also on the overall consistency between predicted and ground truth regions. The final formula for the main loss is as follows:


$${ℒ}_{main}={ℒ}_{dice}+{ℒ}_{ce}$$ (13)

This paper selects cross-entropy loss as the auxiliary loss ${ℒ}_{aux}$ and applies it to the output of the auxiliary head. The auxiliary head takes the features fused from three GLTB modules as input and constructs a 3 × 3 convolution layer with batch normalization and ReLU, a 1 × 1 convolution layer and an upsampling operation to generate the output, which are used to compute the auxiliary loss. To better integrate with the main loss, the auxiliary loss ${ℒ}_{aux}$ is further multiplied by a factor *α*. The final total loss ℒ is the sum of the main loss and the weighted auxiliary loss, as shown in the following formula:


$$ℒ={ℒ}_{main}+\alpha \cdot {ℒ}_{aux}$$ (14)

In the experiments of this paper, *α* is set to 0.4 through cross-dataset ablation studies. The purpose of introducing the auxiliary loss is to provide additional gradient information by supervising the intermediate layer outputs, which helps optimize the GLTB module in the network, thereby improving the overall segmentation performance.

## 4. Experimental results and analyses

### 4.1. Datasets

The experiments in this paper were conducted on three public datasets and a self-constructed dataset: Potsdam [50], Vaihingen [50], UAVid [51] and MSIDBG. For ease of processing and model training, the images were divided into patches of size 1024 × 1024 and 512 × 512 pixels.

Potsdam: The Potsdam dataset is a high-resolution remote sensing imagery dataset specifically designed for semantic segmentation tasks, consisting of 38 aerial images. The original image dimensions are all 6000 × 6000 pixels. This dataset finely categorizes land cover into six classes: impervious surface, building, low vegetation, tree, car, and background (clutter). The dataset contains two main types of data: true orthophotos (True Orthophoto, TOP) with different channel combinations (such as infrared-red-green [IR-R-G], red-green-blue [R-G-B], and red-green-blue-infrared [R-G-B-IR]), and a single-band digital surface model (Digital Surface Model, DSM). The ground sampling distance (GSD) between TOP and DSM is 5 cm, ensuring extremely high spatial resolution. These TOP images are all extracted from a larger mosaic and generated through DSM. In our experiments, we only used the TOP images and their corresponding labels, without boundary information. We used IDs: 2_13, 2_14, 3_13, 3_14, 4_13, 4_14, 4_15, 5_13, 5_14, 5_15, 6_13, 6_14, 6_15, 7_13 as the test set, and the remaining 23 images (excluding image 7_10 with incorrect annotations) were used for training.

Vaihingen: The Vaihingen dataset consists of 33 high-resolution images with a pixel resolution of 0.5 m, sharing the same categories as Potsdam. The images vary in size, averaging 2494 × 2064 pixels, with a GSD of 9 cm. The dataset comprises a TOP covering three bands (near-infrared, red, and green) and a DSM with a single band. In our experiments, we only used the TOP images. We used IDs: 2, 4, 6, 8, 10, 12, 14, 16, 20, 22, 24, 27, 29, 31, 33, 35, 38 as the test set, and the remaining 16 images were used for training.

UAVid: The UAVid dataset is a high-resolution dataset specifically designed for semantic segmentation research, with its uniqueness lying in its focus on images captured by drones in urban environments. The dataset contains 42 video sequences, from which 420 images have been extracted. These images have two high spatial resolutions: 3840 × 2160 pixels and 4096 × 2160 pixels. The image sequences were captured at different urban locations, covering rich land cover categories including building, road, tree, low vegetation, moving car, static car, human, and clutter. The UAVid dataset not only provides top-down views of urban scenes but also includes side views, thus offering more comprehensive and multi-perspective information for object recognition tasks. In our experiments, 200 images were used for training, 70 for validation, and the officially provided 150 images were used for testing.

MSIDBG: The Mangrove Species Identification Dataset in Beihai of Guangxi (MSIDBG) was constructed by us using a DJI Mavic series UAV. Although this dataset uses RGB three-band images, which have less spectral information compared to hyperspectral images, it is easier to collect and more cost-effective. Specifically, the dataset covers mangrove plant communities (Kandelia obovate, Sonneratia apetala, Avicennia marina, Bruguiera gymnorhiza, and Rhizophora stylosa) within the Shankou Mangrove National Nature Reserve in Beihai, Guangxi, China. Each original image has a resolution of 5280 × 3956 pixels. The dataset was annotated manually using the LabelMe tool under the guidance of mangrove experts. To meet the input requirements of deep learning models, selected image areas were uniformly cropped into 512 × 512 pixel. These patches were randomly divided into a training set of 1584 image patches (60%), with both the validation and test sets containing 528 image patches each (20% respectively).

Mangrove species identification faces multiple technical challenges. Different tree species are highly similar in external morphology, resulting in low visual feature distinctiveness, which places higher demands on the network’s ability to extract and discriminate fine-grained features. Simultaneously, target trees often occupy a small proportion in the images, making them prone to missed detection or mis-segmentation, especially under complex background interference. Additionally, mangroves typically grow in dynamic environments such as intertidal zones and swamps, where lighting conditions are complex and variable, often affected by direct sunlight, water surface reflections, and other factors, further increasing the difficulty of stable identification.

### 4.2. Experimental setting

The experimental environment for this paper is as follows: Ubuntu 18.04 operating system, with training and testing conducted on a server equipped with an NVIDIA GeForce RTX3090 GPU (24GB memory). The programming language used is Python 3.8, and the deep learning framework is PyTorch 2.1.1 + cu118. When using the AdamW optimizer, the learning rates for the backbone network and the rest of the model are set to 1 × 10^−3^ and 9 × 10^−4^, respectively, with a weight decay of 0.01 and a batch size of 8. During training, we applied data augmentation techniques including random rotation, vertical and horizontal flipping, random brightness and contrast adjustments, cropping, resizing, and sharpening. For the Potsdam, Vaihingen and MSIDBG datasets, we used random crops of 512 × 512 as input and trained the model for 45, 105 and 100 epochs, respectively, with a batch size of 8 and 4. For the UAVid dataset, we used 1024 × 1024 images as input and trained the model for 40 epochs with a batch size of 8.

### 4.3. Evaluation measure

To evaluate the accuracy of the model, we use F1 score, OA, and mean Intersection over Union (mIoU) as performance metrics on the four datasets mentioned above. The calculation formulas are as follows:


$$P=\frac{\text{1}}{N}\sum_{k=1}^{N}\frac{TP_{k}}{TP_{k}+FP_{k}}$$ (15)

$$R=\frac{\text{1}}{N}\sum_{k=1}^{N}\frac{TP_{k}}{TP_{k}+FN_{k}}$$ (16)

$$F1=2\times \frac{P\times R}{P+R}$$ (17)

$$OA=\frac{\text{1}}{N}\sum_{k=1}^{N}\frac{TP_{k}+TN_{k}}{TP_{k}+FP_{k}+TN_{k}+FN_{k}}$$ (18)

$$mIoU=\frac{\text{1}}{N}\sum_{k=1}^{N}\frac{TP_{k}}{TP_{k}+FP_{k}+FN_{k}}$$ (19)

where 𝑇𝑃_𝑘_, 𝐹𝑃_𝑘_, 𝑇𝑁_𝑘_, and 𝐹𝑁_𝑘_ represent the true positive, false positive, true negative, and false negative for the specific object of class *k*. *P* and *R* denote precision and recall, respectively.

### 4.4. Comparative experiments and analysis

To evaluate the performance of channel reconstruction and dual attention dynamic fusion network (CRDFNet), it was compared with various typical and recent segmentation methods on the Potsdam, Vaihingen, UAVid and MSIDBG datasets. These include three attention-based multiscale aggregation networks: MANet [46], A2-FPN [43], BANet [47], and four methods combining CNNs and transformer: UNetFormer [26], CMTFNet [42], MIFNet [52], and AFENet [45]. In the experimental results table, bold fonts indicate the best data, and the data with “_” are the second-best data.

Table 1 shows the comparative experimental results for the Potsdam dataset. Our proposed CRDFNet achieved the best overall performance with mean F1 of 93.20%, mIoU of 87.48%, and OA of 92.02%, which are 0.37%, 0.62%, and 0.28% higher than the second-best method, respectively. In terms of IoU for each category, CRDFNet also achieved the best results: 89.25% for impervious surface, 94.36% for building, 79.04% for low vegetation, 81.50% for tree, and 93.23% for car. Specifically, for the easily confused categories of impervious surface and building, CRDFNet outperformed the second-best AFENet [45] method by 0.17% and 0.31%, respectively. For the easily confused categories of low vegetation and tree, CRDFNet outperformed the second-best AFENet and MIFNet [52] methods by 0.83% and 0.72%, respectively. This indicates that CRDFNet can better capture global and local context and multiscale features, demonstrating excellent overall performance.

**Table 1:** Segmentation results of different models on the Potsdam dataset.

Methods	Per-Class IoU/F1 Score(%)					mF1(%)	OA(%)	mIoU(%)
	ImpSurf	Building	LowVeg	Tree	Car			
MANet [46]	85.47/92.16	90.31/94.91	75.96/86.34	78.41/87.90	91.63/95.63	91.39	89.69	84.35
A2-FPN [43]	88.34/93.81	92.86/96.30	77.62/87.40	79.61/88.64	91.99/95.83	92.40	91.21	86.08
BANet [47]	86.97/93.03	91.68/95.66	76.62/86.76	79.00/88.27	90.74/95.14	91.77	90.52	85.00
UNetFormer [26]	88.32/93.80	92.82/96.27	77.80/87.51	80.01/88.90	92.72/96.22	92.54	91.29	86.33
CMTFNet [42]	88.74/94.03	93.49/96.64	77.78/87.50	79.84/88.79	93.22/96.49	92.69	91.47	86.62
MIFNet [52]	88.57/93.94	93.48/96.63	78.11/87.93	80.78/89.37	93.14/96.17	92.81	91.43	86.81
AFENet [45]	89.08/**94.39**	94.05/96.77	78.21/87.78	79.75/88.74	93.19/96.47	92.83	91.74	86.86
Ours	**89.25**/94.32	**94.36**/**97.10**	**79.04**/**88.29**	**81.50**/**89.81**	**93.23**/**96.50**	**93.20**	**92.02**	**87.48**

**Fig 5:**
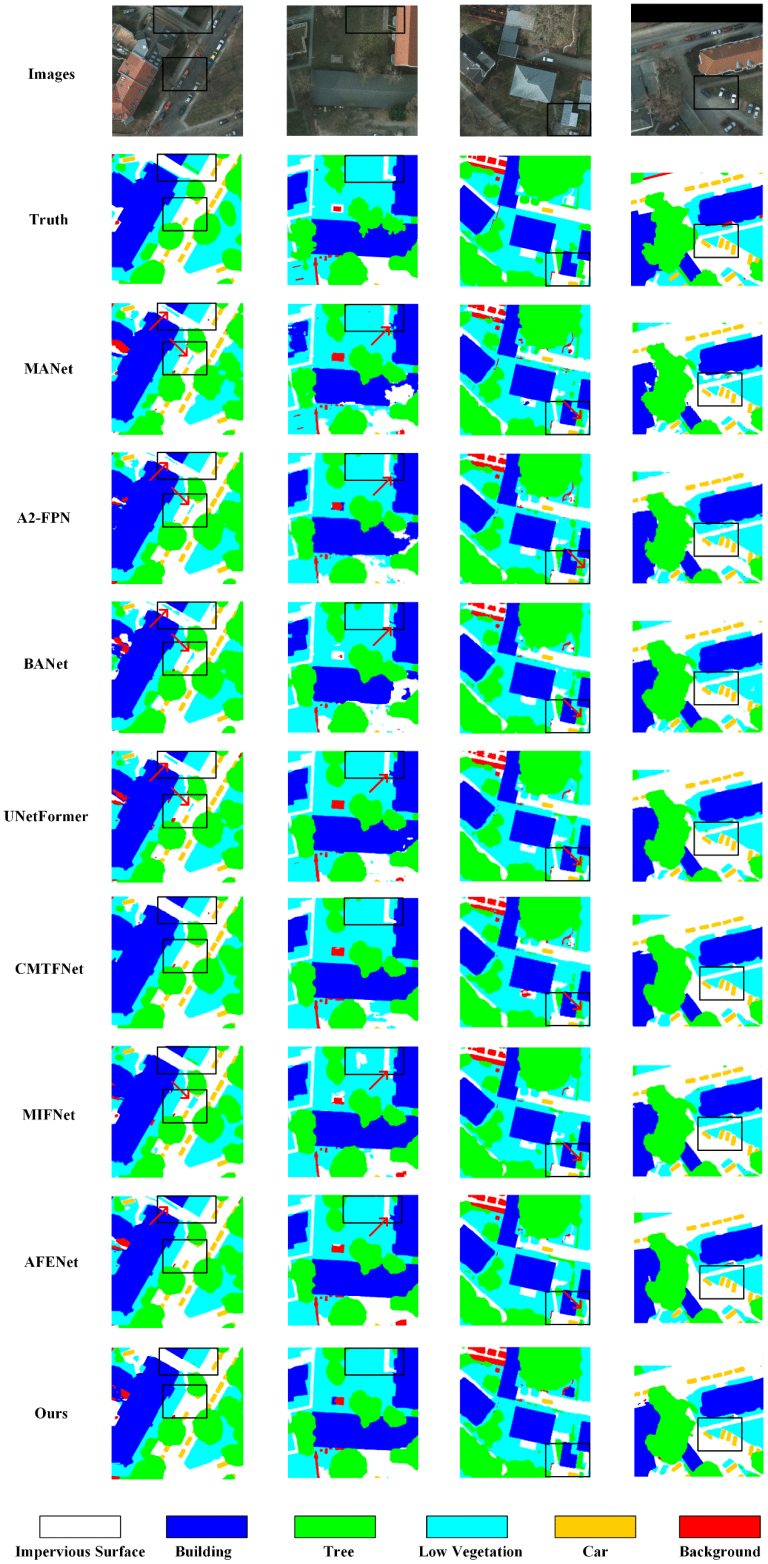
Visualization results of comparative experiments on the Potsdam dataset. The red arrow points to the misidentified regions.

Fig 5 shows the performance of CRDFNet on the Potsdam dataset. The first two columns demonstrate CRDFNet’s advantages in distinguishing impervious surface, building, and low vegetation. Compared to CRDFNet, all other methods except CMTFNet exhibited varying degrees of misidentification. The third column highlights CRDFNet’s superior boundary accuracy and category distinction between tree and impervious surface in complex scenes, while MANet [46], UNetFormer [26], MIFNet [52], and AFENet [45] incorrectly classify impervious surface as low vegetation. In the fourth column, CRDFNet demonstrates more refined boundary segmentation results, showing greater precision in impervious surface segmentation compared to other methods, maintaining clear boundaries and consistency between categories.

Similarly, CRDFNet was evaluated alongside other segmentation models on the Vaihingen dataset, with comparative experimental results shown in Table 2. CRDFNet demonstrated strong overall performance, achieving an mF1 score of 91.95%, an OA score of 93.72%, and an mIoU score of 85.38%, surpassing most existing advanced methods. UNetFormer [26] uses ResNet to extract local features and further utilizes transformer to extract global context, thereby enabling long-range dependency modeling. In contrast, our method employs RegNet to extract local features and integrates them with the channel feature aggregation module (CFAM) to effectively aggregate multiscale features and fuse them with the global context extracted by transformer. As a result, our method shows significant improvements over UNetFormer, with mF1 increasing by 0.87%, OA by 0.27%, and mIoU by 1.42%. Among these improvements, our method introduces a dual attention feature refinement module (DAFRM) that effectively combines shallow spatial information with deep semantic information, achieving precise car category segmentation with an IoU increase of 0.72% over the second-best MIFNet [52], highlighting our method’s excellent performance in small object segmentation.

**Table 2:** Segmentation results of different models on the Vaihingen dataset.

Methods	Per-Class IoU/F1 Score(%)					mF1(%)	OA(%)	mIoU(%)
	ImpSurf	Building	LowVeg	Tree	Car			
MANet [46]	93.30/96.54	89.65/94.52	72.37/83.97	81.93/90.07	77.09/87.07	90.44	92.83	82.87
A2-FPN [43]	94.10/96.96	91.87/95.76	73.71/84.87	82.24/90.25	80.26/89.05	91.38	93.44	84.44
BANet [47]	93.07/96.41	90.39/94.95	72.79/84.25	81.85/90.02	76.04/86.39	90.40	92.90	82.83
UNetFormer [26]	94.07/96.94	92.03/95.85	73.34/84.62	82.18/90.22	78.19/87.76	91.08	93.45	83.96
CMTFNet [42]	94.01/96.91	92.30/95.99	73.88/84.98	82.06/90.15	81.26/89.66	91.54	93.48	84.70
MIFNet [52]	94.06/96.93	92.47/96.09	73.43/84.68	82.25/90.26	82.11/90.18	91.63	93.48	84.86
AFENet [45]	94.09/96.96	92.44/96.07	73.55/84.76	82.06/90.14	80.80/89.38	91.46	93.50	84.58
Ours	**94.34**/**97.09**	**92.61**/**96.16**	**74.66**/**85.49**	**82.47**/**90.31**	**82.83**/**90.61**	**91.95**	**93.72**	**85.38**

**Fig 6:**
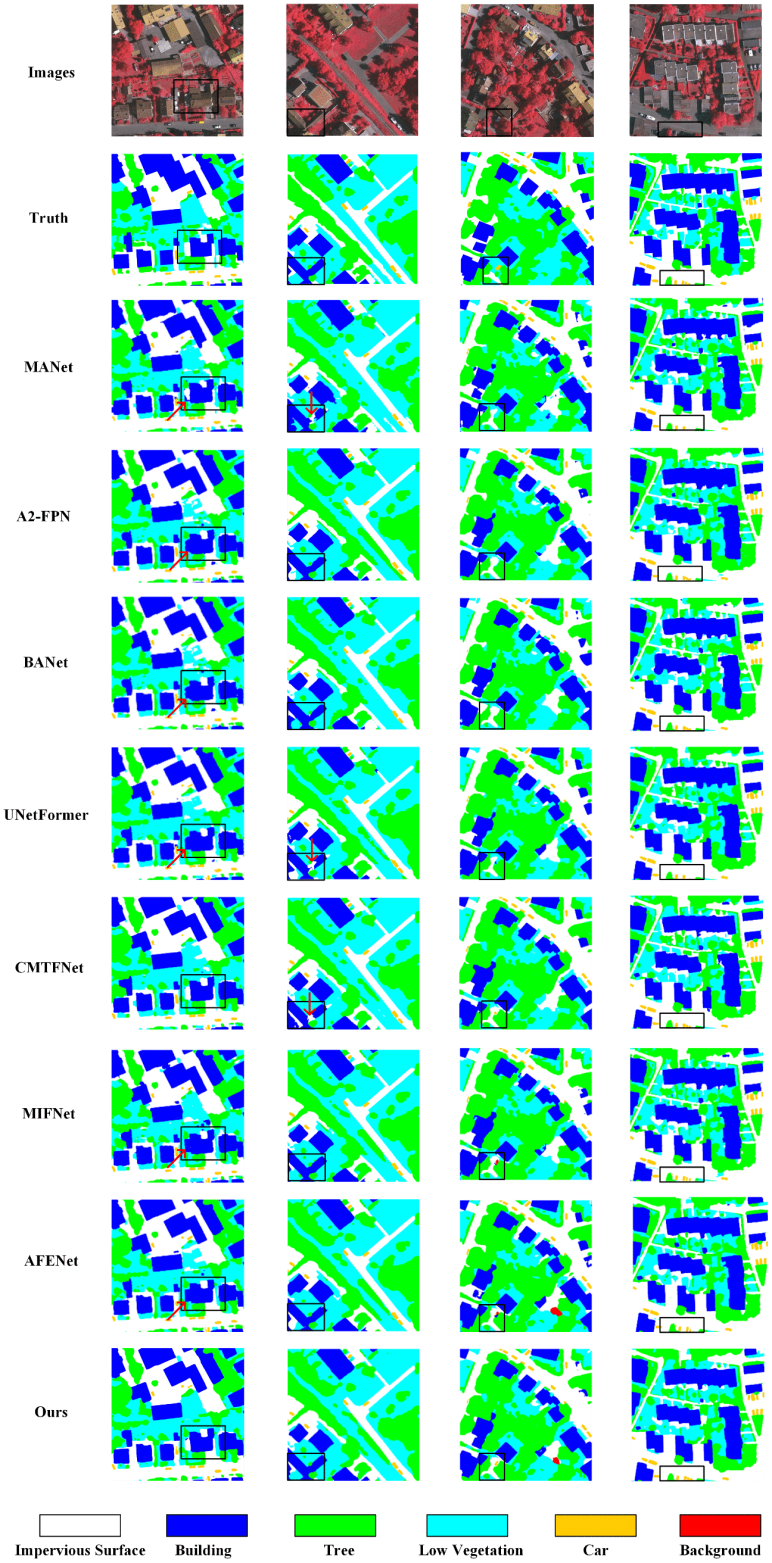
Visualization results of comparative experiments on the Vaihingen dataset. The red arrow points to the misidentified regions.

Fig 6 shows the performance of CRDFNet on the Vaihingen dataset. The first and second columns indicate that CRDFNet clearly delineates the boundaries of buildings while preserving their complete structural shape, and can also distinguish the subtle differences between low vegetation and trees. In contrast, other methods commonly suffer from blurred segmentation boundaries in building segmentation, particularly confusing impervious surface with buildings. The third and fourth columns showcase CRDFNet’s excellent performance in small object segmentation. In the third column, only CRDFNet correctly identifies cars, while in the fourth column, CRDFNet, CMTFNet [42], and MIFNet [52] can more accurately segment cars, whereas MANet [46], UNetFormer [26], and AFENet [45] fail to correctly identify the car category. In summary, CRDFNet can capture subtle category differences in complex scenes, resulting in more coherent and precise segmentation results.

Table 3 shows the comparative experimental results of CRDFNet on the UAVid dataset with other methods. Our method achieved an mIoU of 74.44%, an mF1 score of 84.86%, and an OA of 89.18%, which are 1.30%, 0.89%, and 0.57% higher than the second-best MIFNet [52], respectively. MIFNet performs well in small object segmentation, with the car and human categories achieving second-best results, thanks to its unique multi-dimensional information fusion mechanism. Specifically, MIFNet captures small object features from multiple angles by combining local features, global information, and frequency information. The introduction of frequency information helps the model extract the edges and texture features of small objects in complex backgrounds, while the fusion of local features and global information ensures semantic consistency for small objects. This multi-dimensional information fusion enables MIFNet to maintain high precision and robustness when handling small object segmentation. However, our method also performs excellently, surpassing MIFNet’s IoU by 1.57%, 2.97%, and 1.19% for moving car, static car, and human, respectively. These results fully demonstrate CRDFNet’s outstanding small object recognition ability and precise segmentation capability for irregular shapes in complex scenes.

**Table 3:** Segmentation results of different models on the UAVid dataset.

Methods	Per-Class IoU/F1 Score(%)								mF1(%)	OA(%)	mIoU(%)
	Building	Road	Tree	LowVeg	Mov Car	Static Car	Human	Clutter			
MANet [46]	90.42/94.97	74.53/85.40	77.89/87.57	67.48/80.58	71.52/83.39	68.90/81.59	47.85/64.73	61.12/75.87	81.76	86.73	69.96
A2-FPN [43]	91.67/95.65	78.39/87.89	79.01/88.28	69.93/82.30	71.71/83.52	69.82/82.23	49.64/66.35	65.44/79.11	83.17	88.18	71.95
BANet [47]	90.52/95.02	77.23/87.15	78.21/87.77	67.73/80.76	69.96/82.33	65.69/79.29	40.27/57.41	63.61/77.76	80.94	87.28	69.15
UNetFormer [26]	91.45/95.53	77.06/87.05	78.59/88.01	69.93/82.30	72.93/84.34	69.58/82.06	47.91/64.79	64.28/78.26	82.79	87.85	71.47
CMTFNet [42]	92.19/95.94	80.01/88.90	78.68/88.07	69.68/82.13	73.79/84.92	71.95/83.68	48.66/65.46	66.91/80.18	83.66	88.47	72.73
MIFNet [52]	92.14/94.91	79.25/88.43	79.55/88.61	70.16/82.47	74.65/85.49	72.46/84.03	50.16/66.81	66.74/80.05	83.97	88.61	73.14
AFENet [45]	92.44/96.07	78.78/88.13	79.18/88.38	69.97/82.33	71.62/83.46	69.78/82.20	48.29/65.13	66.88/80.15	83.23	88.54	72.12
Ours	**92.90**/**96.32**	**80.49**/**89.19**	**80.00**/**88.89**	**71.08**/**83.09**	**76.22**/**86.50**	**75.43**/**85.99**	**51.35**/**67.86**	**68.07**/**81.01**	**84.86**	**89.18**	**74.44**

**Fig 7:**
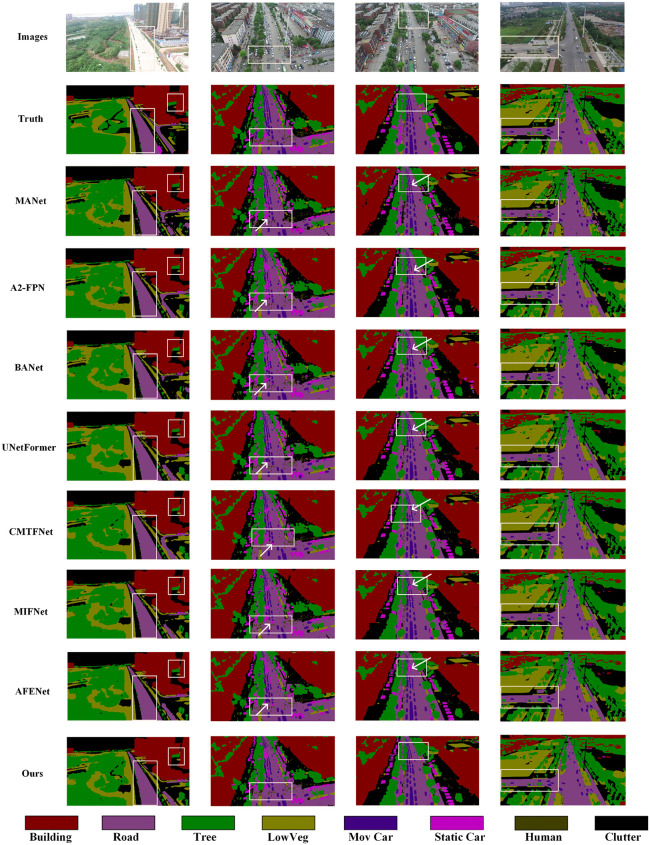
Visualization results of comparative experiments on the UAVid dataset. The white arrow points to the misidentified regions.

Fig 7 shows the segmentation performance of CRDFNet on the UAVid dataset. The first column indicates that CRDFNet can clearly segment the boundaries between the tree and road categories, whereas MANet [46], BANet [47], and MIFNet [52] show poor performance in road boundary segmentation. The second and third columns highlight CRDFNet’s precise segmentation between moving and static cars, while other methods misclassify moving cars as static ones. This demonstrates that CRDFNet has higher segmentation accuracy when handling different states of the same category object. This is mainly attributed to RegNet’s ability to adaptively capture features at different scales by linearly increasing the width and depth of each layer, which helps in segmenting dynamic targets. The fourth column showcases CRDFNet’s excellent performance in segmenting the clutter category, where our model can segment the clutter category more completely. Overall, CRDFNet’s segmentation performance outperforms most existing models, demonstrating its advantages in general segmentation tasks.

Table 4 presents the comparative experimental results of CRDFNet and other methods on the MSIDBG dataset. In this dataset, following its design principles, the background class is ignored and excluded from the calculation of mIoU and mF1, in order to focus more precisely on the mangrove species segmentation task. Experimental results show that our method achieves 89.89% mIoU, 94.61% mF1, and 94.23% OA, which are 1.23%, 0.72%, and 0.34% higher than those of the second-best method, MIFNet [52], respectively. At the class level, Sonneratia apetala and Avicennia marina are prone to confusion under low-resolution or poor lighting conditions due to their similar leaf shapes and colors—particularly, Avicennia marina is often misclassified as Sonneratia apetala. However, CRDFNet shows a significantly better performance on Avicennia marina, with its IoU being 2.89% higher than that of AFENet [45], the second-best method, and 14.31% higher than MANet [46], the lowest-performing one. These results demonstrate that CRDFNet can accurately delineate species boundaries and effectively distinguish between visually similar species in complex scenes, thereby achieving more precise mangrove species segmentation.

**Table 4:** Segmentation results of different models on the MSIDBG dataset.

Methods	Per-Class IoU/F1 Score(%)					mF1(%)	OA(%)	mIoU(%)
	K. obovata	S. apetala	A. marina	B. gymnorhiza	R. stylosa			
MANet [46]	78.39/88.60	84.91/91.32	72.47/83.57	91.22/95.49	93.57/96.68	91.13	93.03	84.05
A2-FPN [43]	82.74/90.55	88.21/93.73	72.50/84.06	**91.97**/**95.82**	94.22/97.02	92.24	93.74	85.93
BANet [47]	82.31/90.30	89.33/94.36	77.50/87.13	91.50/95.56	94.01/**97.07**	92.88	93.86	86.93
UNetFormer [26]	82.18/90.22	87.75/93.48	79.23/88.41	90.96/95.27	93.32/96.54	92.78	93.44	86.69
CMTFNet [42]	81.52/89.82	90.92/95.24	77.42/87.27	91.47/95.55	94.06/96.94	92.96	93.68	87.08
MIFNet [52]	83.75/91.16	91.69/**95.72**	82.59/90.41	91.07/95.32	94.19/97.01	93.92	93.89	88.66
AFENet [45]	81.89/90.05	89.67/94.55	83.89/91.24	91.53/95.58	93.39/96.58	93.60	93.62	88.07
Ours	**85.44**/**92.15**	**91.71**/95.68	**86.78**/**92.92**	91.24/95.42	**94.26**/97.05	**94.64**	**94.23**	**89.89**

**Fig 8:**
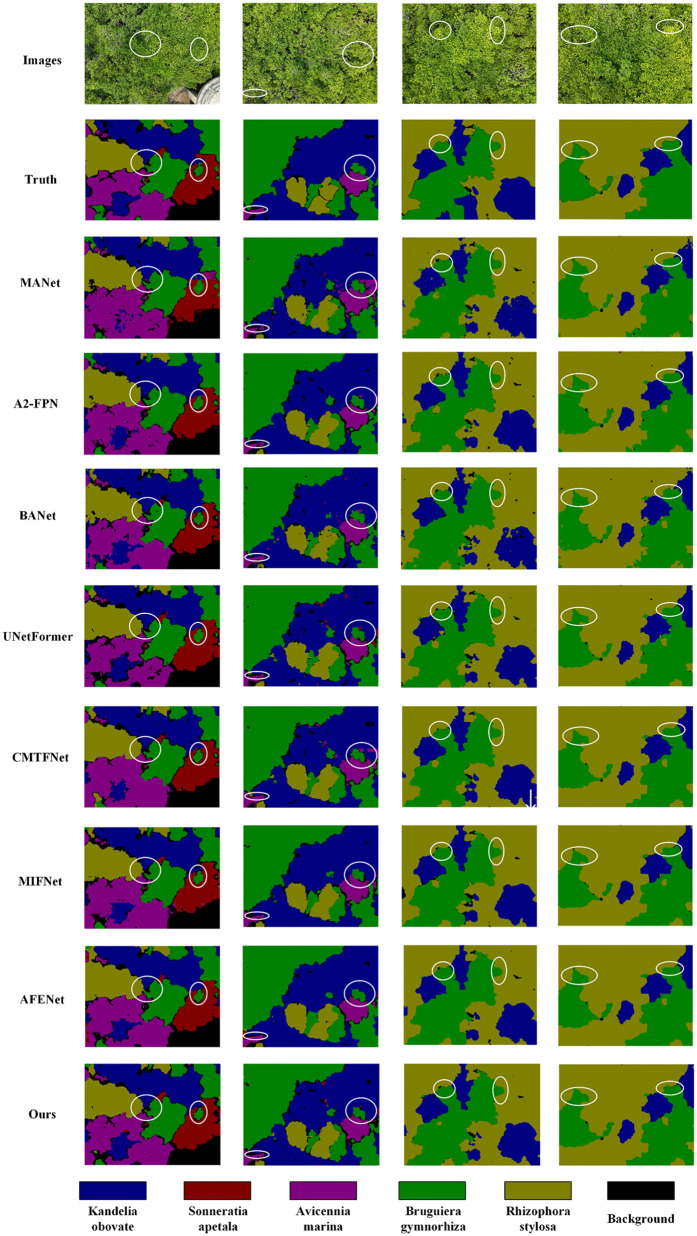
Visualization results of comparative experiments on the MSIDBG dataset.

Fig 8 presents the visualization results of CRDFNet on the MSIDBG dataset. Due to the high morphological similarity among mangrove species, achieving precise segmentation is particularly challenging, especially in overlapping areas, as shown in the first and second columns. In these complex scenes, CRDFNet is able to more accurately identify the boundaries between different species. In the second column, CRDFNet demonstrates strong recognition capability for small target trees such as Avicennia marina. Compared to methods like MANet [46], A2-FPN [43], UNetFormer [26], and MIFNet [52], it significantly alleviates boundary blur issues and achieves clearer segmentation of small targets. Other methods, such as MANet, A2-FPN, BANet [47], and CMTFNet [42], exhibit varying degrees of misclassification—for instance, misidentifying Kandelia obovata as Avicennia marina. The third and fourth columns further illustrate that CRDFNet exhibits robust recognition and segmentation capabilities for the species Bruguiera gymnorhiza and Rhizophora stylosa, effectively improving classification accuracy and mitigating challenges posed by interspecies similarity. Overall, CRDFNet demonstrates remarkable advantages in mangrove species identification, capturing subtle inter-class differences in complex environments and achieving more precise segmentation results.

### 4.5. Ablation experiments and analysis

To verify the effectiveness of CRDFNet, we conducted ablation studies on the Potsdam, Vaihingen, UAVid and MSIDBG datasets to evaluate the contribution of each module to overall performance. In the ablation study, we used UNetFormer [26] as the baseline and analyzed and compared the impact of each module on segmentation performance by replacing the backbone and adding channel feature aggregation module (CFAM) and dual attention feature refinement module (DAFRM).

Tables 5–8 show the ablation experiment results for the Potsdam, Vaihingen, UAVid and MSIDBG datasets. Table 5 shows that, compared to ResNet, RegNet performs better as a backbone in remote sensing image segmentation tasks. RegNet can more effectively build segmentation models for complex scenes by optimizing the network structure design, achieving more refined multiscale feature extraction. The proposed CFAM and DAFRM both show improvements compared to the baseline. Specifically, after replacing the backbone and adding CFAM, the mF1, OA, and mIoU scores improve by 0.43%, 0.56%, and 0.73%, respectively, compared to the baseline. Adding DAFRM improves the scores by 0.45%, 0.52%, and 0.77%. Finally, combining both modules results in improvements of 0.74%, 0.73%, and 1.15% compared to the baseline. Tables 6–8 follow the same pattern as Table 5, with both CFAM and DAFRM showing improvements compared to the baseline. When both modules are added, mF1, OA, and mIoU scores increase by 0.87%, 0.27%, and 1.42%; 2.07%, 1.33%, and 2.97%; and 1.84%, 0.79%, and 3.20% respectively.

**Table 5:** Ablation experiments on the Potsdam dataset.

Methods	mF1(%)	OA(%)	mIoU(%)
Baseline	92.54	91.29	86.33
Baseline+regNet16	92.64	91.65	86.47
Baseline+regNet16 + CFAM	92.97	91.85	87.06
Baseline+regNet16 + DAFRM	92.99	91.81	87.10
Baseline+regNet16 + CFAM+ DAFRM	93.20	92.02	87.48

**Table 6:** Ablation experiments on the Vaihingen dataset.

Methods	mF1(%)	OA(%)	mIoU(%)
Baseline	91.08	93.45	83.96
Baseline+regNet16	91.33	93.22	84.35
Baseline+regNet16 + CFAM	91.77	93.50	85.08
Baseline+regNet16 + DAFRM	91.75	93.50	85.05
Baseline+regNet16 + CFAM+ DAFRM	91.95	93.72	85.38

**Table 7:** Ablation experiments on the UAVid dataset.

Methods	mF1(%)	OA(%)	mIoU(%)
Baseline	82.79	87.85	71.47
Baseline+regNet16	84.15	88.78	73.55
Baseline+regNet16 + CFAM	84.53	88.85	73.94
Baseline+regNet16 + DAFRM	84.58	88.84	74.01
Baseline+regNet16 + CFAM+ DAFRM	84.86	89.18	74.44

**Table 8:** Ablation experiments on the MSIDBG dataset.

Methods	mF1(%)	OA(%)	mIoU(%)
Baseline	92.78	93.44	86.69
Baseline+regNet16	93.71	93.87	88.31
Baseline+regNet16 + CFAM	94.19	94.15	89.13
Baseline+regNet16 + DAFRM	94.23	94.20	89.20
Baseline+regNet16 + CFAM+ DAFRM	94.64	94.23	89.89

**Fig 9:**
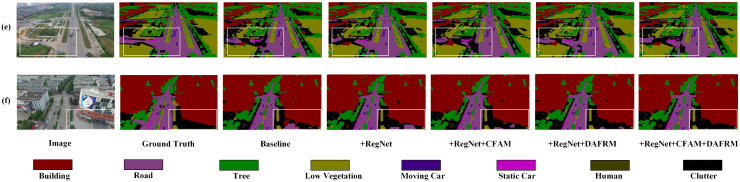
Visualization results of ablation experiments on the UAVid dataset.

**Fig 10:**
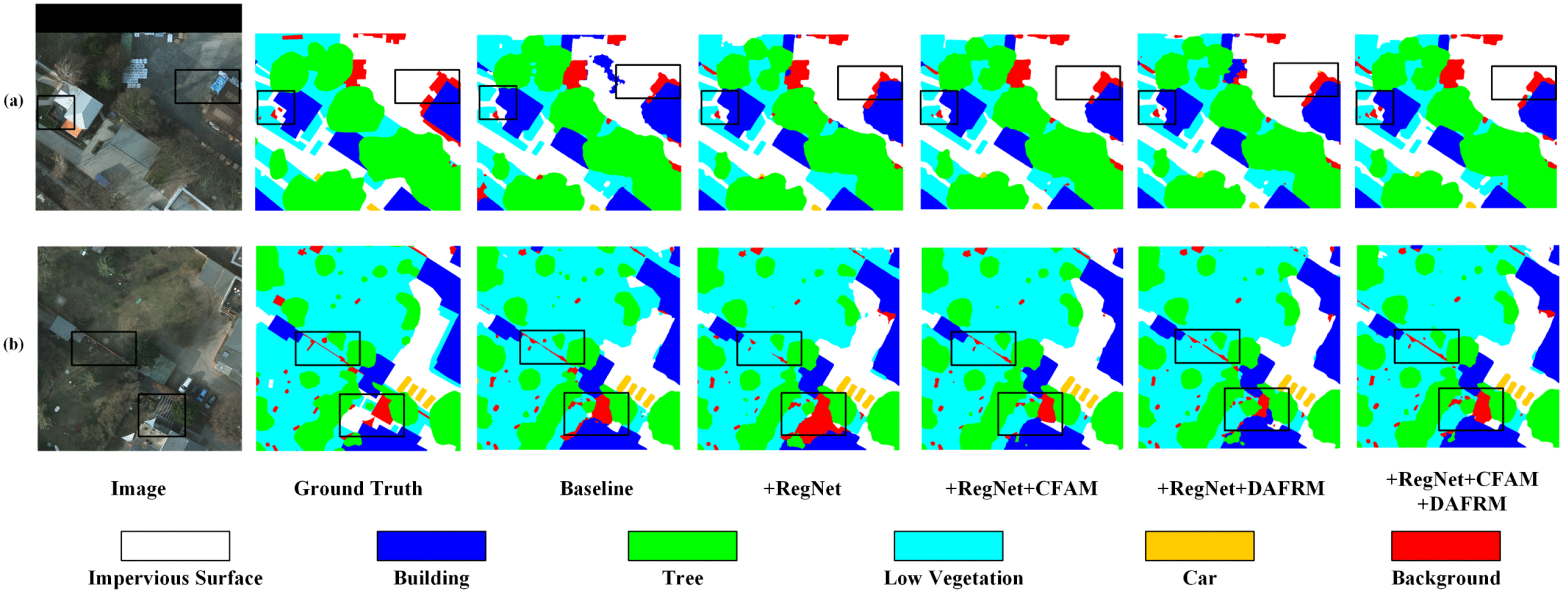
Visualization results of ablation experiments on the Potsdam dataset.

**Fig 11:**
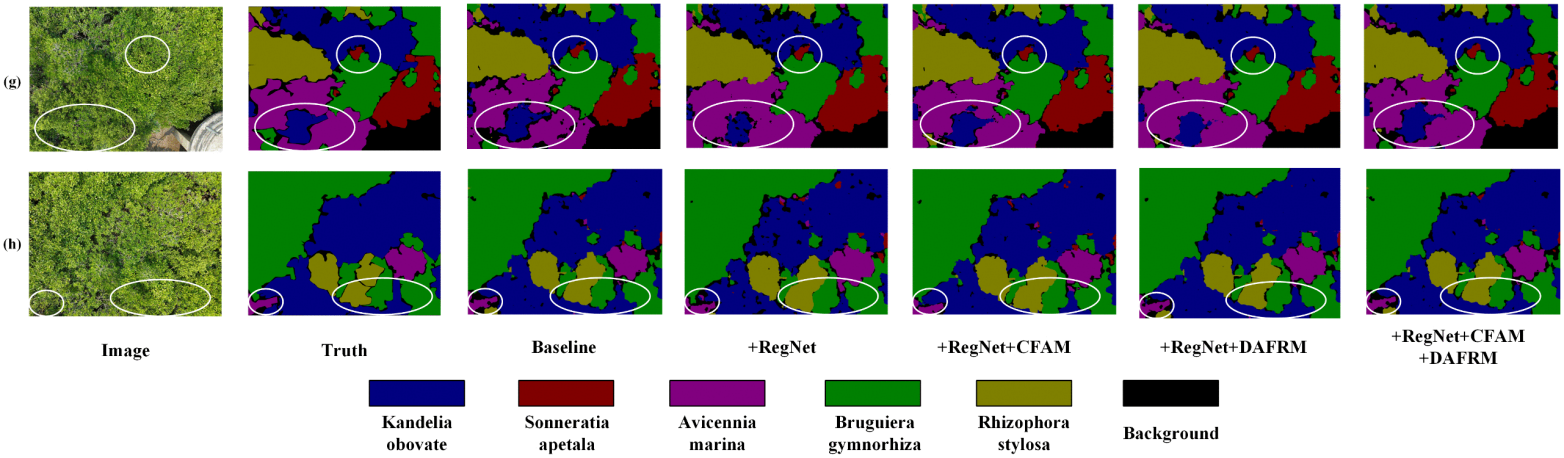
Visualization results of ablation experiments on the MSIDBG dataset.

**Fig 12:**
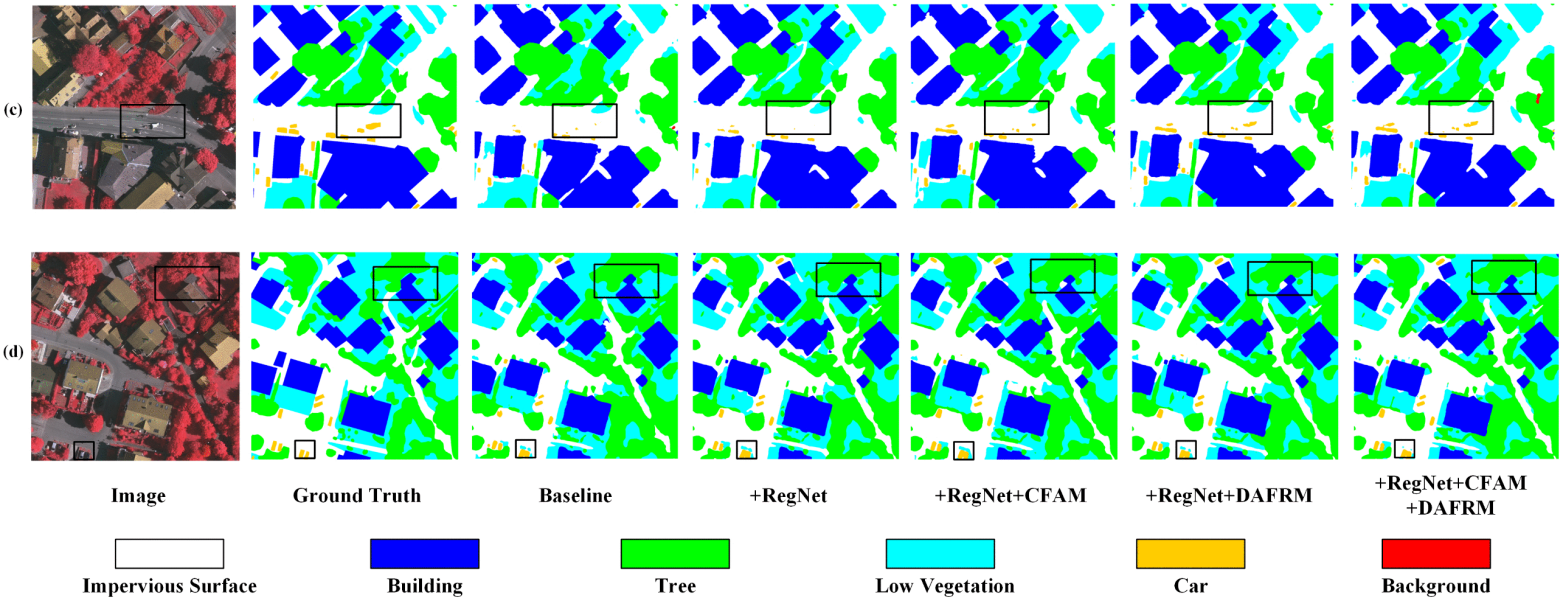
Visualization results of ablation experiments on the Vaihingen dataset.

To more intuitively verify the effectiveness of the proposed modules in this paper, we conducted visual analysis of the effects of individual modules and their combinations. Using RegNet as the backbone, compared to the baseline, it can more effectively capture detailed information in remote sensing images (see (e) in Fig 9). Specifically, CFAM demonstrates significantly better segmentation performance in complex scenes than other modules (as shown in (b) of Fig 10), especially in background category segmentation, while other modules have varying degrees of limitations. To address the similarity among mangrove species, the channel reconstruction mechanism of the CFAM module plays a crucial role. Instead of simply fusing features, it dynamically calibrates and enhances subtle channel features from the encoder that are effective in distinguishing Kandelia obovata and Avicennia marina through a learnable scaling factor $\gamma_{c}$ (as shown in (g) of Fig 11). This mechanism suppresses ineffective features, thereby increasing the distance between similar categories in the feature space. DAFRM shows clear advantages in small object segmentation, particularly in the car category (see (c) in Fig 12) and for small target trees such as Sonneratia apetala (as shown in (g) of Fig 11) and Avicennia marina (as shown in (h) of Fig 11). DAFRM significantly improves segmentation accuracy and target detail capture capability by effectively extracting and fusing shallow features (spatial details) and deep features (semantic information) from images, which is crucial for precise small object segmentation. In summary, the experimental results fully validate the effectiveness of our proposed modules and network structure.

Table 9 presents the ablation results for the auxiliary loss weight α. The experiments show that the model achieves the best performance across all four datasets when α = 0.4. Compared to not using the auxiliary loss (α = 0.0), setting an appropriate α value consistently improves model performance, validating the effective supervisory role of the auxiliary loss in intermediate-layer feature learning. More importantly, the performance exhibits notable robustness within a broad range of α ∈ [0.2, 0.6], with fluctuations remaining minor. This indicates that our multi-loss design is not overly sensitive to this hyperparameter, which is a desirable property for practical applications. While α = 0.4 is identified as the consistent peak, the marginal variation in this range further confirms that the auxiliary loss acts as a stable and complementary guide to the primary loss, rather than dominantly steering the optimization. The performance decline observed at α = 0.8 on most datasets (Potsdam, Vaihingen, and MSIDBG) reinforces that an appropriate balance is key, an excessively strong auxiliary signal can begin to interfere with the primary training objective.

**Table 9:** Ablation study on the weight α mIoU (%) was measured for the Potsdam, Vaihingen, UAVid and MSIDBG dataset.

Dataset	α				
	0.0	0.2	0.4	0.6	0.8
Potsdam	87.07	87.25	87.48	87.12	86.94
Vaihingen	84.87	84.93	85.38	85.12	85.07
UAVid	73.81	73.90	74.44	73.95	74.10
MSIDBG	88.88	89.28	89.89	89.27	89.03

### 4.6. Model complexity analysis

We compared multiple CNN-based encoders on the Potsdam dataset (as shown in Table 10). The experiments demonstrate that RegNet achieves the highest segmentation accuracy (87.48% mIoU) while maintaining the lowest or near-lowest parameter count (9.4M) and computational cost (12.04G FLOPs). This advantage stems from RegNet’s automatically designed network architecture based on quantized linear rules, which more efficiently aligns with the feature extraction requirements of remote sensing images compared to manually designed networks like ResNet, achieving a better balance in depth and width. Therefore, we select RegNet as the encoder backbone for CRDFNet, aiming to obtain the strongest feature representation capability with the minimal computational overhead, thereby providing high-quality multi-scale feature foundations for the subsequent CFAM and DAFRM modules.

**Table 10:** FLOPs, Params, and mIoU of each encoder-based segmentation model on the Potsdam dataset.

Dataset	Backbone	FLOPs(G)	Params(M)	mIoU(%)
Potsdam	ResNet18	13.24	12.31	86.30
	ResNet50	24.92	24.59	86.44
	ResNet101	44.42	43.59	87.19
	EfficientNet_B3	8.50	11.12	87.27
	RegNet	12.04	9.40	87.48

Table 11 below evaluates the model complexity of CRDFNet using two metrics, the number of floating-point operation counts (FLOPs) and model parameters (Params). It is worth noting that the FLOPs and Params for all methods are based on an image that is 512 × 512 and uses a single NVIDIA GeForce RTX3090 GPU. CRDFNet achieves the lowest Params (9.40M) among all compared methods, which is approximately 19.5% lower than that of UNetFormer. The computational cost (12.04G FLOPs) is comparable to the most efficient model, UNetFormer, but significantly lower than other models based on complex attention mechanisms or multi-dimensional fusion, such as MANet and MIFNet.

**Table 11:** Computational complexity analysis on a single NVIDIA GeForce RTX3090 GPU.

Methods	FLOPs(G)	Params(M)
MANet	77.68	35.86
A2-FPN	41.83	22.82
BANet	13.06	12.73
UNetFormer	11.74	11.68
CMTFNet	33.07	30.07
MIFNet	35.42	25.35
AFENet	25.59	20.23
Ours	12.04	9.40

## 5. Conclusions

In this paper, we propose channel reconstruction and dual attention dynamic fusion network (CRDFNet), a remote sensing image semantic segmentation model that effectively fuses global and local context information. Our model achieves high-quality segmentation results by combining a CNN-based efficient encoder and a transformer-based decoder, with channel feature aggregation module (CFAM) serving as a bridge between them. Additionally, the introduction of dual attention feature refinement module (DAFRM) enables the model to perform excellently in small object segmentation. Experimental results show that CRDFNet demonstrates high accuracy when processing complex high-resolution images and achieves excellent performance on four datasets: Potsdam, Vaihingen, UAVid, and MSIDBG. Furthermore, by comparing with other models in terms of complexity, we prove that CRDFNet can provide superior segmentation performance while maintaining low computational costs. Future research will continue to explore the application potential of CRDFNet in high-resolution remote sensing image semantic segmentation and attempt to introduce more innovative attention mechanisms and more efficient adaptive feature fusion strategies, ensuring that the network further improves the efficiency of remote sensing image segmentation while balancing category sensitivity and segmentation equilibrium.
